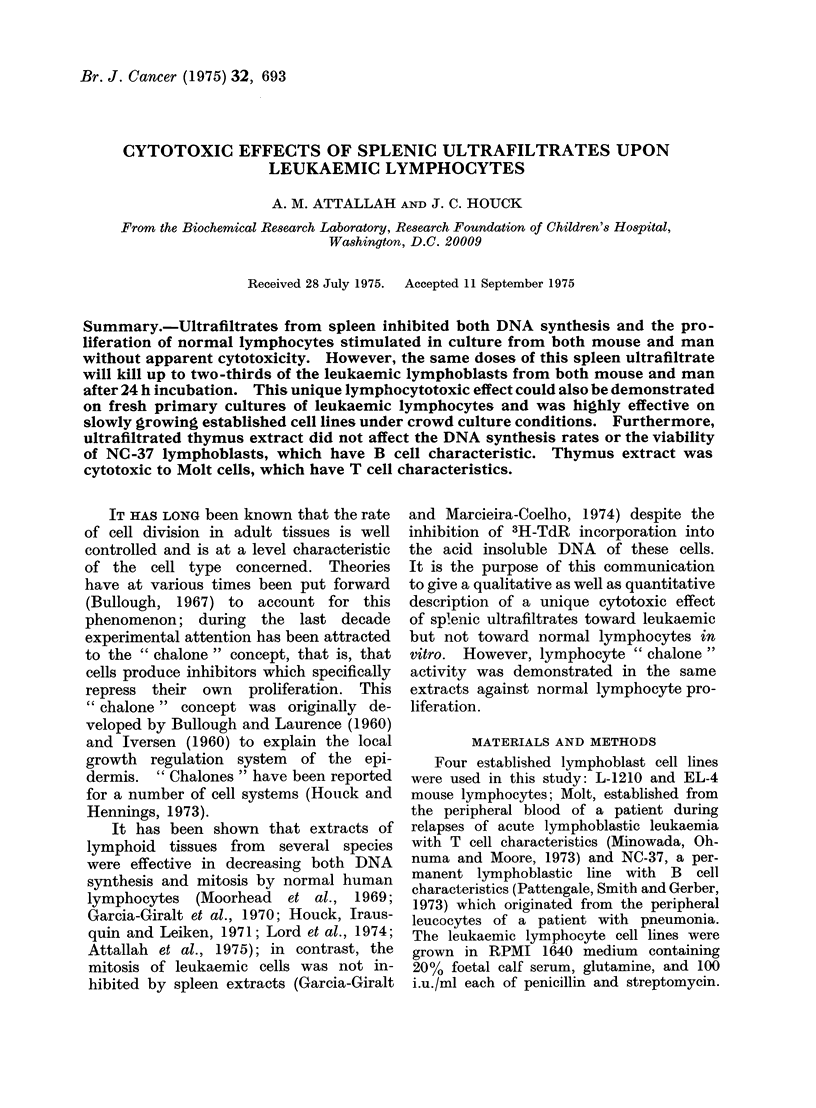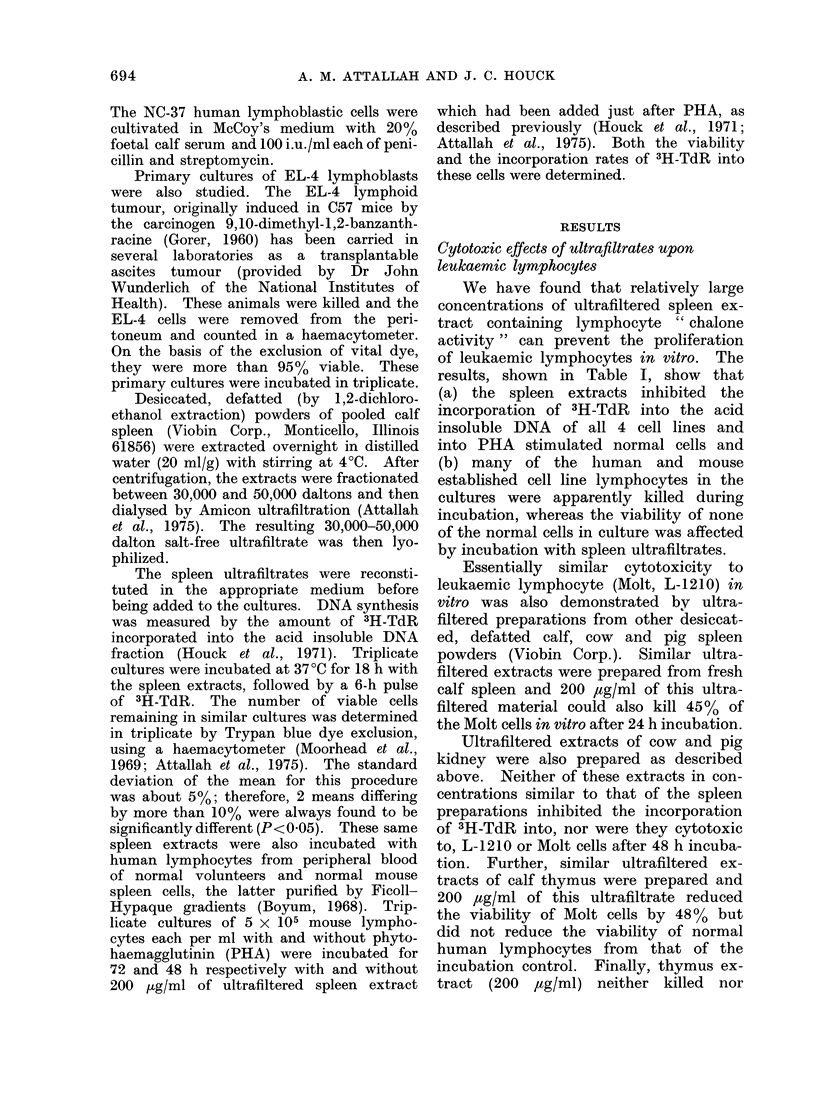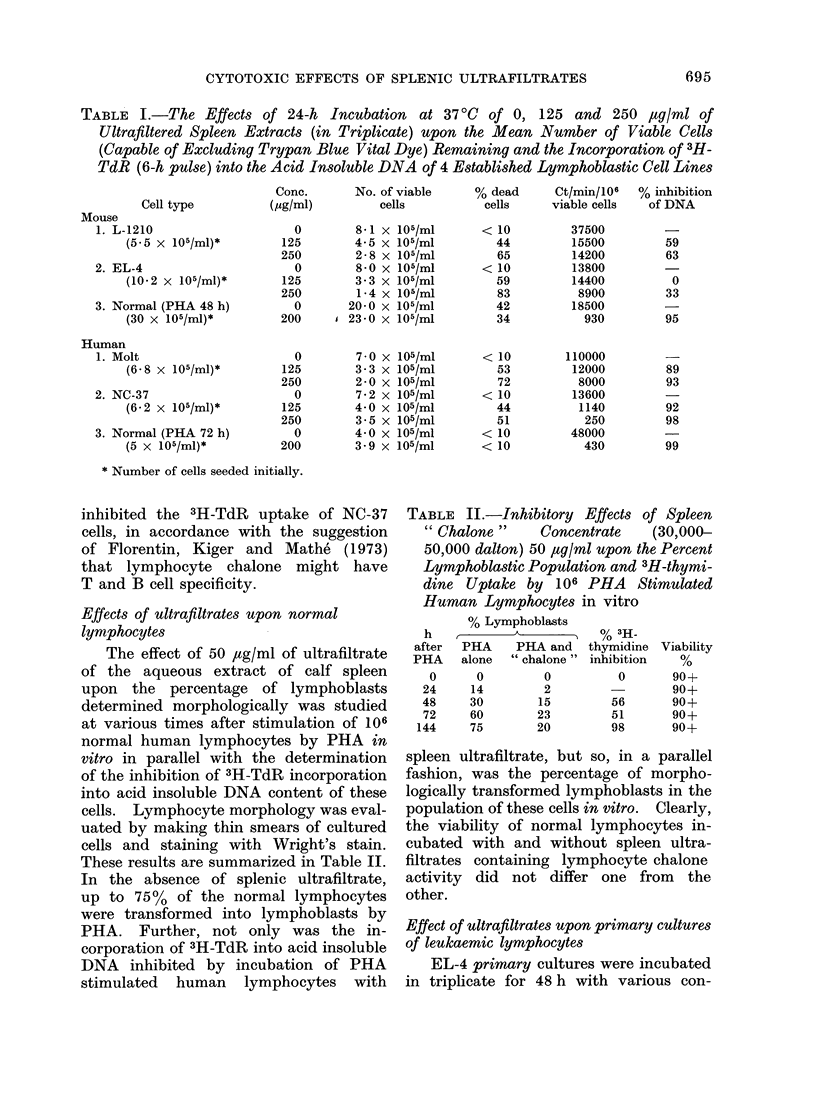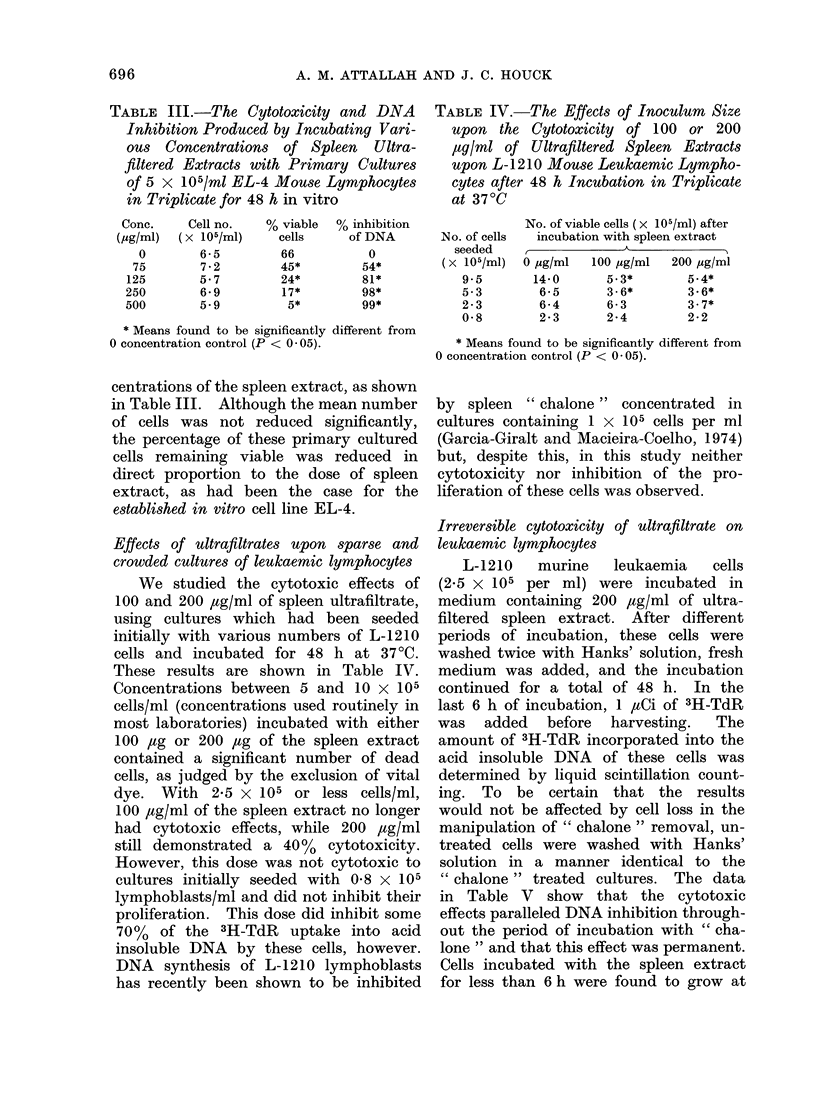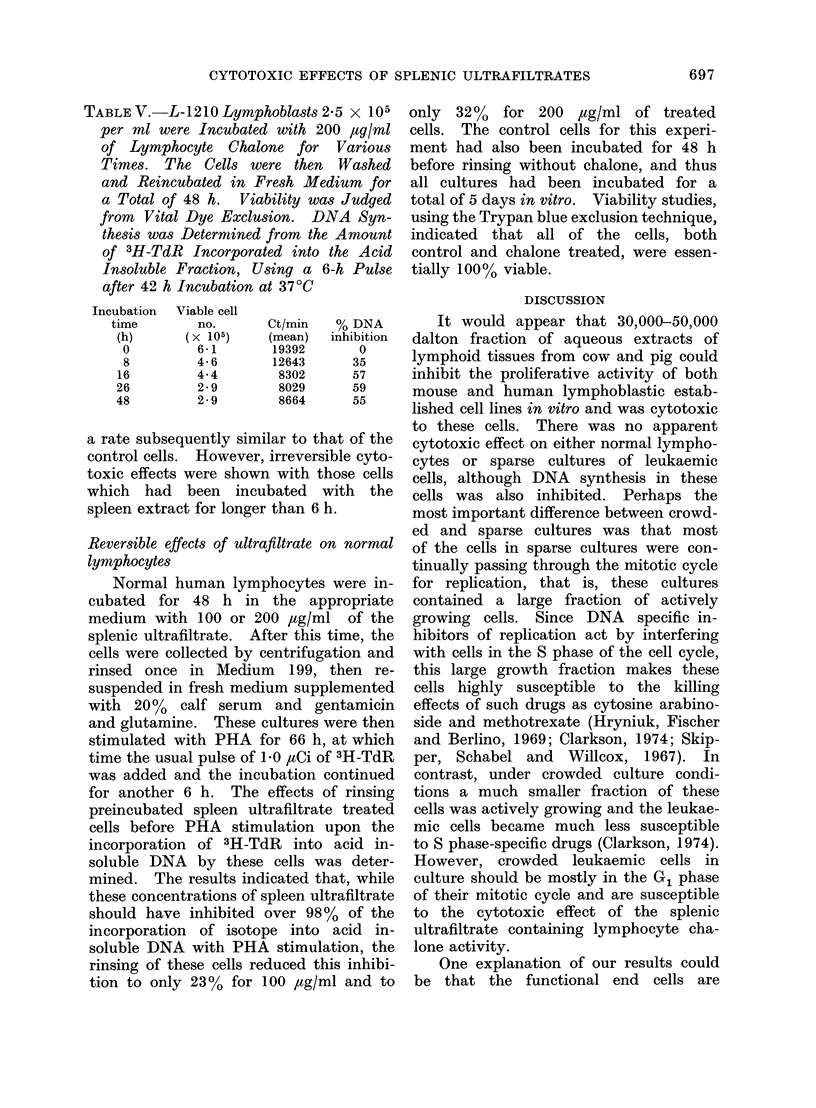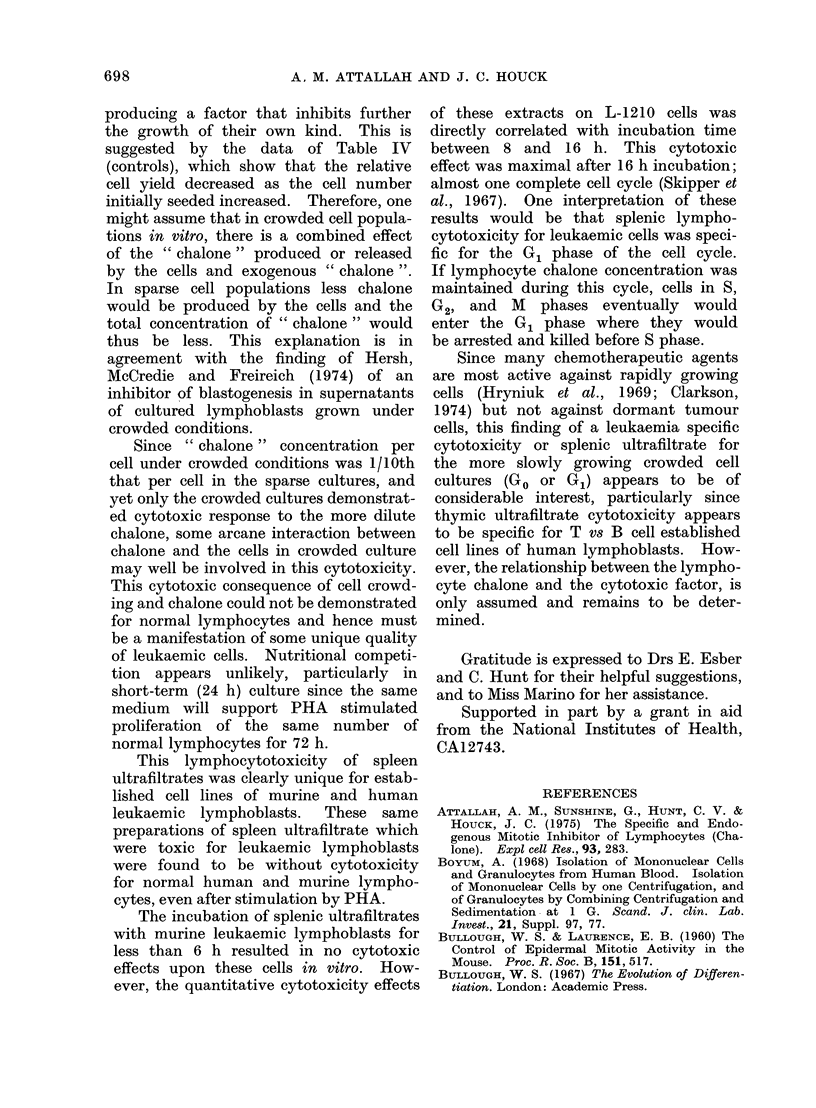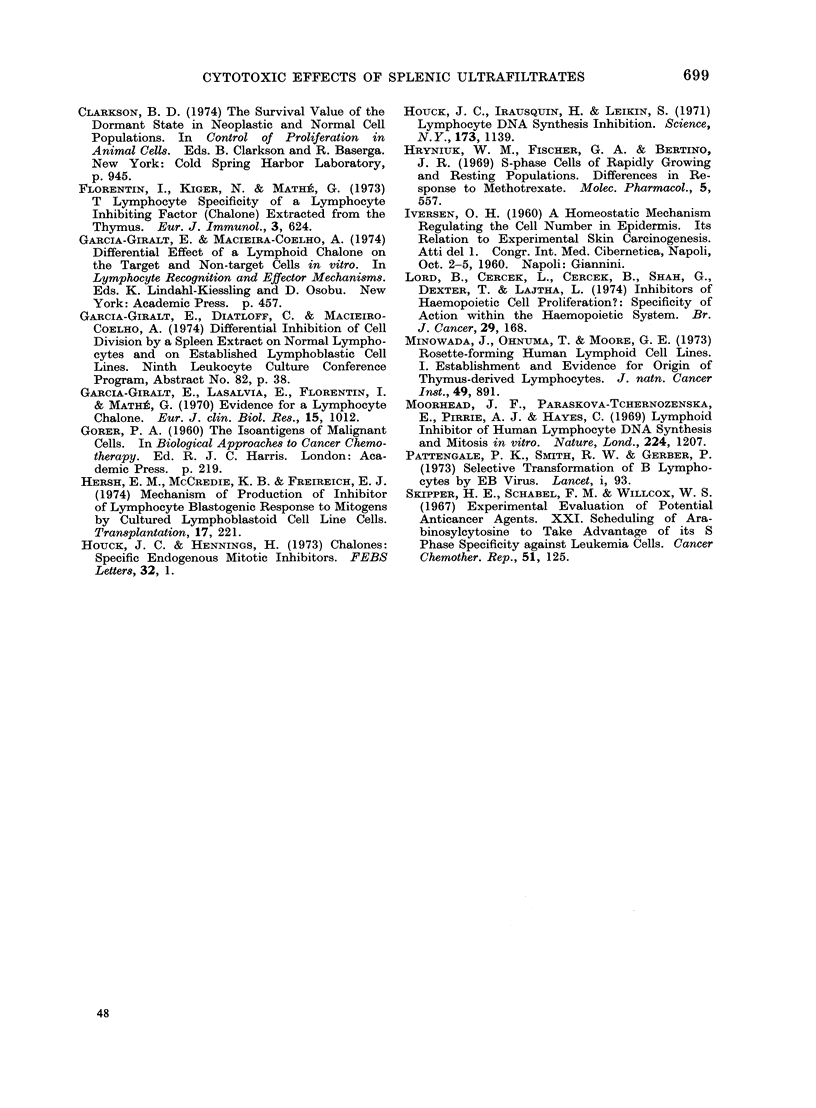# Cytotoxic effects on splenic ultrafiltrates upon leukaemic lymphocytes.

**DOI:** 10.1038/bjc.1975.280

**Published:** 1975-12

**Authors:** A. M. Attallah, J. C. Houck

## Abstract

Ultrafiltrates from spleen inhibited both DNA synthesis and the proliferation of normal lymphocytes stimulated inculture from both mouse and man without apparent cytotoxicity. However, the same doses of this spleen ultrafiltrate will kill up to two-thirds of the leukaemic lymphoblasts from both mouse and man after 24 h incubation. This unique lymphocytotoxic effect could also be demonstrated on fresh primary cultures of leukaemic lymphocytes and was highly effective on slowly growing established cell lines under crowd culture conditions. Furthermore. ultrafiltrated thymus extract did not affect the DNA synthesis rates of the viability of NC-37 lymphoblasts, which have B cell characteristic. Thymus extract was cytotoxic to Molt cells, which have T cell characteristics.


*Br. J. Cancer* (1975) **32**, 693

### CYTOTOXIC EFFECTS OF SPLENIC ULTRAFILTRATES UPON LEUKAEMIC LYMPHOCYTES

A. M. ATTALLAH AND J. C. HOUCK

*From the Biochemical Research Laboratory, Research Foundation of Children's Hospital, Washington, D.C. 20009*

Received 28 July 1975. Accepted 11 September 1975

**Summary.—Ultrafiltrates from spleen inhibited both DNA synthesis and the proliferation of normal lymphocytes stimulated in culture from both mouse and man without apparent cytotoxicity. However, the same doses of this spleen ultrafiltrate will kill up to two-thirds of the leukaemic lymphoblasts from both mouse and man after 24 h incubation. This unique lymphocytotoxic effect could also be demonstrated on fresh primary cultures of leukaemic lymphocytes and was highly effective on slowly growing established cell lines under crowd culture conditions. Furthermore, ultrafiltrated thymus extract did not affect the DNA synthesis rates or the viability of NC-37 lymphoblasts, which have B cell characteristic. Thymus extract was cytotoxic to Molt cells, which have T cell characteristics.**

IT HAS LONG been known that the rate of cell division in adult tissues is well controlled and is at a level characteristic of the cell type concerned. Theories have at various times been put forward (Bullough, 1967) to account for this phenomenon; during the last decade experimental attention has been attracted to the " chalone " concept, that is, that cells produce inhibitors which specifically repress their own proliferation. This " chalone " concept was originally developed by Bullough and Laurence (1960) and Iversen (1960) to explain the local growth regulation system of the epidermis. " Chalones " have been reported for a number of cell systems (Houck and Hennings, 1973).

It has been shown that extracts of lymphoid tissues from several species were effective in decreasing both DNA synthesis and mitosis by normal human lymphocytes (Moorhead *et al.*, 1969; Garcia-Giralt *et al.*, 1970; Houck, Irausquin and Leiken, 1971; Lord *et al.*, 1974; Attallah *et al.*, 1975); in contrast, the mitosis of leukaemic cells was not inhibited by spleen extracts (Garcia-Giralt and Marcieira-Coelho, 1974) despite the inhibition of $^3$H-TdR incorporation into the acid insoluble DNA of these cells. It is the purpose of this communication to give a qualitative as well as quantitative description of a unique cytotoxic effect of splenic ultrafiltrates toward leukaemic but not toward normal lymphocytes *in vitro*. However, lymphocyte " chalone " activity was demonstrated in the same extracts against normal lymphocyte proliferation.

### MATERIALS AND METHODS

Four established lymphoblast cell lines were used in this study: L-1210 and EL-4 mouse lymphocytes; Molt, established from the peripheral blood of a patient during relapses of acute lymphoblastic leukaemia with T cell characteristics (Minowada, Ohnuma and Moore, 1973) and NC-37, a permanent lymphoblastic line with B cell characteristics (Pattengale, Smith and Gerber, 1973) which originated from the peripheral leucocytes of a patient with pneumonia. The leukaemic lymphocyte cell lines were grown in RPMI 1640 medium containing 20% foetal calf serum, glutamine, and 100 i.u./ml each of penicillin and streptomycin.

The NC-37 human lymphoblastic cells were cultivated in McCoy's medium with 20% foetal calf serum and 100 i.u./ml each of penicillin and streptomycin.

Primary cultures of EL-4 lymphoblasts were also studied. The EL-4 lymphoid tumour, originally induced in C57 mice by the carcinogen 9,10-dimethyl-1,2-banzanthracine (Gorer, 1960) has been carried in several laboratories as a transplantable ascites tumour (provided by Dr John Wunderlich of the National Institutes of Health). These animals were killed and the EL-4 cells were removed from the peritoneum and counted in a haemacytometer. On the basis of the exclusion of vital dye, they were more than 95% viable. These primary cultures were incubated in triplicate.

Desiccated, defatted (by 1,2-dichloroethanol extraction) powders of pooled calf spleen (Viobin Corp., Monticello, Illinois 61856) were extracted overnight in distilled water (20 ml/g) with stirring at 4°C. After centrifugation, the extracts were fractionated between 30,000 and 50,000 daltons and then dialysed by Amicon ultrafiltration (Attallah *et al.*, 1975). The resulting 30,000–50,000 dalton salt-free ultrafiltrate was then lyophilized.

The spleen ultrafiltrates were reconstituted in the appropriate medium before being added to the cultures. DNA synthesis was measured by the amount of $^3$H-TdR incorporated into the acid insoluble DNA fraction (Houck *et al.*, 1971). Triplicate cultures were incubated at 37°C for 18 h with the spleen extracts, followed by a 6-h pulse of $^3$H-TdR. The number of viable cells remaining in similar cultures was determined in triplicate by Trypan blue dye exclusion, using a haemacytometer (Moorhead *et al.*, 1969; Attallah *et al.*, 1975). The standard deviation of the mean for this procedure was about 5%; therefore, 2 means differing by more than 10% were always found to be significantly different ($P<0{\cdot}05$). These same spleen extracts were also incubated with human lymphocytes from peripheral blood of normal volunteers and normal mouse spleen cells, the latter purified by Ficoll–Hypaque gradients (Boyum, 1968). Triplicate cultures of $5 \times 10^5$ mouse lymphocytes each per ml with and without phytohaemagglutinin (PHA) were incubated for 72 and 48 h respectively with and without 200 $\mu$g/ml of ultrafiltered spleen extract which had been added just after PHA, as described previously (Houck *et al.*, 1971; Attallah *et al.*, 1975). Both the viability and the incorporation rates of $^3$H-TdR into these cells were determined.

### RESULTS

### *Cytotoxic effects of ultrafiltrates upon leukaemic lymphocytes*

We have found that relatively large concentrations of ultrafiltered spleen extract containing lymphocyte "chalone activity" can prevent the proliferation of leukaemic lymphocytes *in vitro*. The results, shown in Table I, show that (a) the spleen extracts inhibited the incorporation of $^3$H-TdR into the acid insoluble DNA of all 4 cell lines and into PHA stimulated normal cells and (b) many of the human and mouse established cell line lymphocytes in the cultures were apparently killed during incubation, whereas the viability of none of the normal cells in culture was affected by incubation with spleen ultrafiltrates.

Essentially similar cytotoxicity to leukaemic lymphocyte (Molt, L-1210) *in vitro* was also demonstrated by ultrafiltered preparations from other desiccated, defatted calf, cow and pig spleen powders (Viobin Corp.). Similar ultrafiltered extracts were prepared from fresh calf spleen and 200 $\mu$g/ml of this ultrafiltered material could also kill 45% of the Molt cells *in vitro* after 24 h incubation.

Ultrafiltered extracts of cow and pig kidney were also prepared as described above. Neither of these extracts in concentrations similar to that of the spleen preparations inhibited the incorporation of $^3$H-TdR into, nor were they cytotoxic to, L-1210 or Molt cells after 48 h incubation. Further, similar ultrafiltered extracts of calf thymus were prepared and 200 $\mu$g/ml of this ultrafiltrate reduced the viability of Molt cells by 48% but did not reduce the viability of normal human lymphocytes from that of the incubation control. Finally, thymus extract (200 $\mu$g/ml) neither killed nor

TABLE I.—*The Effects of 24-h Incubation at 37°C of 0, 125 and 250 μg/ml of Ultrafiltered Spleen Extracts (in Triplicate) upon the Mean Number of Viable Cells (Capable of Excluding Trypan Blue Vital Dye) Remaining and the Incorporation of $^3$H-TdR (6-h pulse) into the Acid Insoluble DNA of* 4 *Established Lymphoblastic Cell Lines*

| Cell type | Conc. (μg/ml) | No. of viable cells | % dead cells | Ct/min/$10^6$ viable cells | % inhibition of DNA |
|---|---|---|---|---|---|
| Mouse | | | | | |
| 1. L-1210 | 0 | $8{\cdot}1 \times 10^5$/ml | < 10 | 37500 | — |
| ($5{\cdot}5 \times 10^5$/ml)* | 125 | $4{\cdot}5 \times 10^5$/ml | 44 | 15500 | 59 |
| | 250 | $2{\cdot}8 \times 10^5$/ml | 65 | 14200 | 63 |
| 2. EL-4 | 0 | $8{\cdot}0 \times 10^5$/ml | < 10 | 13800 | — |
| ($10{\cdot}2 \times 10^5$/ml)* | 125 | $3{\cdot}3 \times 10^5$/ml | 59 | 14400 | 0 |
| | 250 | $1{\cdot}4 \times 10^5$/ml | 83 | 8900 | 33 |
| 3. Normal (PHA 48 h) | 0 | $20{\cdot}0 \times 10^5$/ml | 42 | 18500 | — |
| ($30 \times 10^5$/ml)* | 200 | $23{\cdot}0 \times 10^5$/ml | 34 | 930 | 95 |
| Human | | | | | |
| 1. Molt | 0 | $7{\cdot}0 \times 10^5$/ml | < 10 | 110000 | — |
| ($6{\cdot}8 \times 10^5$/ml)* | 125 | $3{\cdot}3 \times 10^5$/ml | 53 | 12000 | 89 |
| | 250 | $2{\cdot}0 \times 10^5$/ml | 72 | 8000 | 93 |
| 2. NC-37 | 0 | $7{\cdot}2 \times 10^5$/ml | < 10 | 13600 | — |
| ($6{\cdot}2 \times 10^5$/ml)* | 125 | $4{\cdot}0 \times 10^5$/ml | 44 | 1140 | 92 |
| | 250 | $3{\cdot}5 \times 10^5$/ml | 51 | 250 | 98 |
| 3. Normal (PHA 72 h) | 0 | $4{\cdot}0 \times 10^5$/ml | < 10 | 48000 | — |
| ($5 \times 10^5$/ml)* | 200 | $3{\cdot}9 \times 10^5$/ml | < 10 | 430 | 99 |

* Number of cells seeded initially.

inhibited the $^3$H-TdR uptake of NC-37 cells, in accordance with the suggestion of Florentin, Kiger and Mathé (1973) that lymphocyte chalone might have T and B cell specificity.

*Effects of ultrafiltrates upon normal lymphocytes*

The effect of 50 μg/ml of ultrafiltrate of the aqueous extract of calf spleen upon the percentage of lymphoblasts determined morphologically was studied at various times after stimulation of $10^6$ normal human lymphocytes by PHA *in vitro* in parallel with the determination of the inhibition of $^3$H-TdR incorporation into acid insoluble DNA content of these cells. Lymphocyte morphology was evaluated by making thin smears of cultured cells and staining with Wright's stain. These results are summarized in Table II. In the absence of splenic ultrafiltrate, up to 75% of the normal lymphocytes were transformed into lymphoblasts by PHA. Further, not only was the incorporation of $^3$H-TdR into acid insoluble DNA inhibited by incubation of PHA stimulated human lymphocytes with

TABLE II.—*Inhibitory Effects of Spleen "Chalone" Concentrate (30,000–50,000 dalton) 50 μg/ml upon the Percent Lymphoblastic Population and $^3$H-thymidine Uptake by $10^6$ PHA Stimulated Human Lymphocytes* in vitro

| h after PHA | % Lymphoblasts: PHA alone | % Lymphoblasts: PHA and "chalone" | % $^3$H-thymidine inhibition | Viability % |
|---|---|---|---|---|
| 0 | 0 | 0 | 0 | 90+ |
| 24 | 14 | 2 | — | 90+ |
| 48 | 30 | 15 | 56 | 90+ |
| 72 | 60 | 23 | 51 | 90+ |
| 144 | 75 | 20 | 98 | 90+ |

spleen ultrafiltrate, but so, in a parallel fashion, was the percentage of morphologically transformed lymphoblasts in the population of these cells *in vitro*. Clearly, the viability of normal lymphocytes incubated with and without spleen ultrafiltrates containing lymphocyte chalone activity did not differ one from the other.

*Effect of ultrafiltrates upon primary cultures of leukaemic lymphocytes*

EL-4 *primary* cultures were incubated in triplicate for 48 h with various con-

TABLE III.—*The Cytotoxicity and DNA Inhibition Produced by Incubating Various Concentrations of Spleen Ultrafiltered Extracts with Primary Cultures of* 5 × $10^5$*/ml EL*-4 *Mouse Lymphocytes in Triplicate for* 48 *h* in vitro

| Conc. (μg/ml) | Cell no. (× $10^5$/ml) | % viable cells | % inhibition of DNA |
|---|---|---|---|
| 0 | 6·5 | 66 | 0 |
| 75 | 7·2 | 45* | 54* |
| 125 | 5·7 | 24* | 81* |
| 250 | 6·9 | 17* | 98* |
| 500 | 5·9 | 5* | 99* |

* Means found to be significantly different from 0 concentration control ($P < 0·05$).

TABLE IV.—*The Effects of Inoculum Size upon the Cytotoxicity of* 100 *or* 200 *μg/ml of Ultrafiltered Spleen Extracts upon L*-1210 *Mouse Leukaemic Lymphocytes after* 48 *h Incubation in Triplicate at* 37°*C*

| No. of cells seeded (× $10^5$/ml) | No. of viable cells (× $10^5$/ml) after incubation with spleen extract | | |
|---|---|---|---|
| | 0 μg/ml | 100 μg/ml | 200 μg/ml |
| 9·5 | 14·0 | 5·3* | 5·4* |
| 5·3 | 6·5 | 3·6* | 3·6* |
| 2·3 | 6·4 | 6·3 | 3·7* |
| 0·8 | 2·3 | 2·4 | 2·2 |

* Means found to be significantly different from 0 concentration control ($P < 0·05$).

centrations of the spleen extract, as shown in Table III. Although the mean number of cells was not reduced significantly, the percentage of these primary cultured cells remaining viable was reduced in direct proportion to the dose of spleen extract, as had been the case for the *established in vitro* cell line EL-4.

*Effects of ultrafiltrates upon sparse and crowded cultures of leukaemic lymphocytes*

We studied the cytotoxic effects of 100 and 200 μg/ml of spleen ultrafiltrate, using cultures which had been seeded initially with various numbers of L-1210 cells and incubated for 48 h at 37°C. These results are shown in Table IV. Concentrations between 5 and 10 × $10^5$ cells/ml (concentrations used routinely in most laboratories) incubated with either 100 μg or 200 μg of the spleen extract contained a significant number of dead cells, as judged by the exclusion of vital dye. With 2·5 × $10^5$ or less cells/ml, 100 μg/ml of the spleen extract no longer had cytotoxic effects, while 200 μg/ml still demonstrated a 40% cytotoxicity. However, this dose was not cytotoxic to cultures initially seeded with 0·8 × $10^5$ lymphoblasts/ml and did not inhibit their proliferation. This dose did inhibit some 70% of the $^3$H-TdR uptake into acid insoluble DNA by these cells, however. DNA synthesis of L-1210 lymphoblasts has recently been shown to be inhibited by spleen " chalone " concentrated in cultures containing 1 × $10^5$ cells per ml (Garcia-Giralt and Macieira-Coelho, 1974) but, despite this, in this study neither cytotoxicity nor inhibition of the proliferation of these cells was observed.

*Irreversible cytotoxicity of ultrafiltrate on leukaemic lymphocytes*

L-1210 murine leukaemia cells (2·5 × $10^5$ per ml) were incubated in medium containing 200 μg/ml of ultrafiltered spleen extract. After different periods of incubation, these cells were washed twice with Hanks' solution, fresh medium was added, and the incubation continued for a total of 48 h. In the last 6 h of incubation, 1 μCi of $^3$H-TdR was added before harvesting. The amount of $^3$H-TdR incorporated into the acid insoluble DNA of these cells was determined by liquid scintillation counting. To be certain that the results would not be affected by cell loss in the manipulation of " chalone " removal, untreated cells were washed with Hanks' solution in a manner identical to the " chalone " treated cultures. The data in Table V show that the cytotoxic effects paralleled DNA inhibition throughout the period of incubation with " chalone " and that this effect was permanent. Cells incubated with the spleen extract for less than 6 h were found to grow at

TABLE V.—*L-1210 Lymphoblasts* $2 \cdot 5 \times 10^5$ *per ml were Incubated with 200* $\mu g/ml$ *of Lymphocyte Chalone for Various Times. The Cells were then Washed and Reincubated in Fresh Medium for a Total of 48 h. Viability was Judged from Vital Dye Exclusion. DNA Synthesis was Determined from the Amount of* $^3H$*-TdR Incorporated into the Acid Insoluble Fraction, Using a 6-h Pulse after 42 h Incubation at 37°C*

| Incubation time (h) | Viable cell no. ($\times 10^5$) | Ct/min (mean) | % DNA inhibition |
|---|---|---|---|
| 0 | 6·1 | 19392 | 0 |
| 8 | 4·6 | 12643 | 35 |
| 16 | 4·4 | 8302 | 57 |
| 26 | 2·9 | 8029 | 59 |
| 48 | 2·9 | 8664 | 55 |

a rate subsequently similar to that of the control cells. However, irreversible cytotoxic effects were shown with those cells which had been incubated with the spleen extract for longer than 6 h.

*Reversible effects of ultrafiltrate on normal lymphocytes*

Normal human lymphocytes were incubated for 48 h in the appropriate medium with 100 or 200 $\mu$g/ml of the splenic ultrafiltrate. After this time, the cells were collected by centrifugation and rinsed once in Medium 199, then resuspended in fresh medium supplemented with 20% calf serum and gentamicin and glutamine. These cultures were then stimulated with PHA for 66 h, at which time the usual pulse of 1·0 $\mu$Ci of $^3$H-TdR was added and the incubation continued for another 6 h. The effects of rinsing preincubated spleen ultrafiltrate treated cells before PHA stimulation upon the incorporation of $^3$H-TdR into acid insoluble DNA by these cells was determined. The results indicated that, while these concentrations of spleen ultrafiltrate should have inhibited over 98% of the incorporation of isotope into acid insoluble DNA with PHA stimulation, the rinsing of these cells reduced this inhibition to only 23% for 100 $\mu$g/ml and to only 32% for 200 $\mu$g/ml of treated cells. The control cells for this experiment had also been incubated for 48 h before rinsing without chalone, and thus all cultures had been incubated for a total of 5 days *in vitro*. Viability studies, using the Trypan blue exclusion technique, indicated that all of the cells, both control and chalone treated, were essentially 100% viable.

### DISCUSSION

It would appear that 30,000–50,000 dalton fraction of aqueous extracts of lymphoid tissues from cow and pig could inhibit the proliferative activity of both mouse and human lymphoblastic established cell lines *in vitro* and was cytotoxic to these cells. There was no apparent cytotoxic effect on either normal lymphocytes or sparse cultures of leukaemic cells, although DNA synthesis in these cells was also inhibited. Perhaps the most important difference between crowded and sparse cultures was that most of the cells in sparse cultures were continually passing through the mitotic cycle for replication, that is, these cultures contained a large fraction of actively growing cells. Since DNA specific inhibitors of replication act by interfering with cells in the S phase of the cell cycle, this large growth fraction makes these cells highly susceptible to the killing effects of such drugs as cytosine arabinoside and methotrexate (Hryniuk, Fischer and Berlino, 1969; Clarkson, 1974; Skipper, Schabel and Willcox, 1967). In contrast, under crowded culture conditions a much smaller fraction of these cells was actively growing and the leukaemic cells became much less susceptible to S phase-specific drugs (Clarkson, 1974). However, crowded leukaemic cells in culture should be mostly in the $G_1$ phase of their mitotic cycle and are susceptible to the cytotoxic effect of the splenic ultrafiltrate containing lymphocyte chalone activity.

One explanation of our results could be that the functional end cells are producing a factor that inhibits further the growth of their own kind. This is suggested by the data of Table IV (controls), which show that the relative cell yield decreased as the cell number initially seeded increased. Therefore, one might assume that in crowded cell populations *in vitro*, there is a combined effect of the " chalone " produced or released by the cells and exogenous " chalone ". In sparse cell populations less chalone would be produced by the cells and the total concentration of " chalone " would thus be less. This explanation is in agreement with the finding of Hersh, McCredie and Freireich (1974) of an inhibitor of blastogenesis in supernatants of cultured lymphoblasts grown under crowded conditions.

Since " chalone " concentration per cell under crowded conditions was 1/10th that per cell in the sparse cultures, and yet only the crowded cultures demonstrated cytotoxic response to the more dilute chalone, some arcane interaction between chalone and the cells in crowded culture may well be involved in this cytotoxicity. This cytotoxic consequence of cell crowding and chalone could not be demonstrated for normal lymphocytes and hence must be a manifestation of some unique quality of leukaemic cells. Nutritional competition appears unlikely, particularly in short-term (24 h) culture since the same medium will support PHA stimulated proliferation of the same number of normal lymphocytes for 72 h.

This lymphocytotoxicity of spleen ultrafiltrates was clearly unique for established cell lines of murine and human leukaemic lymphoblasts. These same preparations of spleen ultrafiltrate which were toxic for leukaemic lymphoblasts were found to be without cytotoxicity for normal human and murine lymphocytes, even after stimulation by PHA.

The incubation of splenic ultrafiltrates with murine leukaemic lymphoblasts for less than 6 h resulted in no cytotoxic effects upon these cells *in vitro*. However, the quantitative cytotoxicity effects of these extracts on L-1210 cells was directly correlated with incubation time between 8 and 16 h. This cytotoxic effect was maximal after 16 h incubation; almost one complete cell cycle (Skipper *et al.*, 1967). One interpretation of these results would be that splenic lymphocytotoxicity for leukaemic cells was specific for the $G_1$ phase of the cell cycle. If lymphocyte chalone concentration was maintained during this cycle, cells in S, $G_2$, and M phases eventually would enter the $G_1$ phase where they would be arrested and killed before S phase.

Since many chemotherapeutic agents are most active against rapidly growing cells (Hryniuk *et al.*, 1969; Clarkson, 1974) but not against dormant tumour cells, this finding of a leukaemia specific cytotoxicity or splenic ultrafiltrate for the more slowly growing crowded cell cultures ($G_0$ or $G_1$) appears to be of considerable interest, particularly since thymic ultrafiltrate cytotoxicity appears to be specific for T *vs* B cell established cell lines of human lymphoblasts. However, the relationship between the lymphocyte chalone and the cytotoxic factor, is only assumed and remains to be determined.

Gratitude is expressed to Drs E. Esber and C. Hunt for their helpful suggestions, and to Miss Marino for her assistance.

Supported in part by a grant in aid from the National Institutes of Health, CA12743.

### REFERENCES

Attallah, A. M., Sunshine, G., Hunt, C. V. & Houck, J. C. (1975) The Specific and Endogenous Mitotic Inhibitor of Lymphocytes (Chalone). *Expl cell Res.*, **93**, 283.

Boyum, A. (1968) Isolation of Mononuclear Cells and Granulocytes from Human Blood. Isolation of Mononuclear Cells by one Centrifugation, and of Granulocytes by Combining Centrifugation and Sedimentation at 1 G. *Scand. J. clin. Lab. Invest.*, **21**, Suppl. 97, 77.

Bullough, W. S. & Laurence, E. B. (1960) The Control of Epidermal Mitotic Activity in the Mouse. *Proc. R. Soc.* B, **151**, 517.

Bullough, W. S. (1967) *The Evolution of Differentiation.* London: Academic Press.

CLARKSON, B. D. (1974) The Survival Value of the Dormant State in Neoplastic and Normal Cell Populations. In *Control of Proliferation in Animal Cells*. Eds. B. Clarkson and R. Baserga. New York: Cold Spring Harbor Laboratory, p. 945.

FLORENTIN, I., KIGER, N. & MATHÉ, G. (1973) T Lymphocyte Specificity of a Lymphocyte Inhibiting Factor (Chalone) Extracted from the Thymus. *Eur. J. Immunol.*, **3**, 624.

GARCIA-GIRALT, E. & MACIEIRA-COELHO, A. (1974) Differential Effect of a Lymphoid Chalone on the Target and Non-target Cells *in vitro*. In *Lymphocyte Recognition and Effector Mechanisms*. Eds. K. Lindahl-Kiessling and D. Osobu. New York: Academic Press. p. 457.

GARCIA-GIRALT, E., DIATLOFF, C. & MACIEIRO-COELHO, A. (1974) Differential Inhibition of Cell Division by a Spleen Extract on Normal Lymphocytes and on Established Lymphoblastic Cell Lines. Ninth Leukocyte Culture Conference Program, Abstract No. 82, p. 38.

GARCIA-GIRALT, E., LASALVIA, E., FLORENTIN, I. & MATHÉ, G. (1970) Evidence for a Lymphocyte Chalone. *Eur. J. clin. Biol. Res.*, **15**, 1012.

GORER, P. A. (1960) The Isoantigens of Malignant Cells. In *Biological Approaches to Cancer Chemotherapy*. Ed. R. J. C. Harris. London: Academic Press. p. 219.

HERSH, E. M., MCCREDIE, K. B. & FREIREICH, E. J. (1974) Mechanism of Production of Inhibitor of Lymphocyte Blastogenic Response to Mitogens by Cultured Lymphoblastoid Cell Line Cells. *Transplantation*, **17**, 221.

HOUCK, J. C. & HENNINGS, H. (1973) Chalones: Specific Endogenous Mitotic Inhibitors. *FEBS Letters*, **32**, 1.

HOUCK, J. C., IRAUSQUIN, H. & LEIKIN, S. (1971) Lymphocyte DNA Synthesis Inhibition. *Science, N.Y.*, **173**, 1139.

HRYNIUK, W. M., FISCHER, G. A. & BERTINO, J. R. (1969) S-phase Cells of Rapidly Growing and Resting Populations. Differences in Response to Methotrexate. *Molec. Pharmacol.*, **5**, 557.

IVERSEN, O. H. (1960) A Homeostatic Mechanism Regulating the Cell Number in Epidermis. Its Relation to Experimental Skin Carcinogenesis. Atti del 1. Congr. Int. Med. Cibernetica, Napoli, Oct. 2–5, 1960. Napoli: Giannini.

LORD, B., CERCEK, L., CERCEK, B., SHAH, G., DEXTER, T. & LAJTHA, L. (1974) Inhibitors of Haemopoietic Cell Proliferation?: Specificity of Action within the Haemopoietic System. *Br. J. Cancer*, **29**, 168.

MINOWADA, J., OHNUMA, T. & MOORE, G. E. (1973) Rosette-forming Human Lymphoid Cell Lines. I. Establishment and Evidence for Origin of Thymus-derived Lymphocytes. *J. natn. Cancer Inst.*, **49**, 891.

MOORHEAD, J. F., PARASKOVA-TCHERNOZENSKA, E., PIRRIE, A. J. & HAYES, C. (1969) Lymphoid Inhibitor of Human Lymphocyte DNA Synthesis and Mitosis *in vitro*. *Nature, Lond.*, **224**, 1207.

PATTENGALE, P. K., SMITH, R. W. & GERBER, P. (1973) Selective Transformation of B Lymphocytes by EB Virus. *Lancet*, i, 93.

SKIPPER, H. E., SCHABEL, F. M. & WILLCOX, W. S. (1967) Experimental Evaluation of Potential Anticancer Agents. XXI. Scheduling of Arabinosylcytosine to Take Advantage of its S Phase Specificity against Leukemia Cells. *Cancer Chemother. Rep.*, **51**, 125.